# Ethyl 2-[(*Z*)-2-benzyl­hydrazin-1-yl­idene]-2-bromo­acetate

**DOI:** 10.1107/S1600536810032071

**Published:** 2010-08-18

**Authors:** Qian-Jiao Yang, Dan Liu, Jian Zuo, Guo-Dong Hu, Lin-Xiang Zhao

**Affiliations:** a Key Laboratory of Original New Drug Design and Discovery of the Ministry of Education, College of Pharmaceutical Engineering, Shenyang Pharmaceutical University, Shenyang, Liaoning 110016, People’s Republic of China; b Key Laboratory of Marine Chemistry Theory and Technology, Ministry of Education, College of Chemistry and Chemical Engineering, Ocean University of China, Qingdao, Shandong 266100, People’s Republic of China

## Abstract

In the title compound, C_11_H_13_BrN_2_O_2_, the dihedral angle between the phenyl ring and the almost planar (r.m.s. deviation = 0.011 Å) C—C(Br)=N—N(H)— fragment is 74.94 (16)°. In the crystal, mol­ecules are linked by N—H⋯O hydrogen bonds, which generate *C*(6) chains propagating in [010]. Weak aromatic π–π stacking [centroid–centroid separation = 3.784 (3) Å] may also help to consolidate the packing.

## Related literature

For the use of the title compound in the preparation of heterocyclic compounds *via* the diploar cyclo­addition of thia­diazole, see Feddouli *et al.* (2004 ▶); Abouricha *et al.* (2005 ▶); Hafez *et al.* (2008 ▶). For the synthesis of the title compound, see Bach *et al.* (1994 ▶).
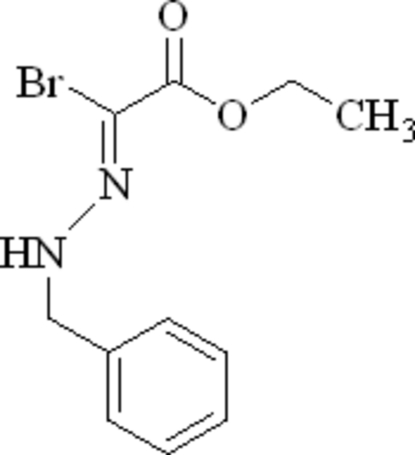


## Experimental

### 

#### Crystal data

C_11_H_13_BrN_2_O_2_
*M* *_r_* = 285.14
Monoclinic, 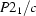

*a* = 9.046 (1) Å
*b* = 11.235 (1) Å
*c* = 12.326 (2) Å
β = 92.935 (4)°
*V* = 1251.1 (3) Å^3^
*Z* = 4
Mo *K*α radiation
μ = 3.27 mm^−1^
*T* = 294 K
0.25 × 0.14 × 0.07 mm

#### Data collection

Siemens APEX CCD diffractometer
Absorption correction: multi-scan (*SADABS*; Siemens, 1996 ▶) *T*_min_ = 0.495, *T*_max_ = 0.803
4952 measured reflections
2188 independent reflections
1475 reflections with *I* > 2σ(*I*)
*R*_int_ = 0.019

#### Refinement

*R*[*F* ^2^ > 2σ(*F* ^2^)] = 0.038
*wR*(*F* ^2^) = 0.111
*S* = 1.02
2188 reflections
146 parameters
H-atom parameters constrained
Δρ_max_ = 0.26 e Å^−3^
Δρ_min_ = −0.61 e Å^−3^

### 

Data collection: *SMART* (Siemens, 1996 ▶); cell refinement: *SAINT* (Siemens, 1996 ▶); data reduction: *SAINT*; program(s) used to solve structure: *SHELXS97* (Sheldrick, 2008 ▶); program(s) used to refine structure: *SHELXL97* (Sheldrick, 2008 ▶); molecular graphics: *SHELXTL* (Sheldrick, 2008 ▶); software used to prepare material for publication: *SHELXTL*.

## Supplementary Material

Crystal structure: contains datablocks I, global. DOI: 10.1107/S1600536810032071/hb5585sup1.cif

Structure factors: contains datablocks I. DOI: 10.1107/S1600536810032071/hb5585Isup2.hkl

Additional supplementary materials:  crystallographic information; 3D view; checkCIF report

## Figures and Tables

**Table 1:** Hydrogen-bond geometry (Å, °)

*D*—H⋯*A*	*D*—H	H⋯*A*	*D*⋯*A*	*D*—H⋯*A*
N1—H1⋯O1^i^	0.86	2.24	2.965 (4)	141

Symmetry code: (i) 
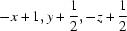
.

**Fig. 1.**
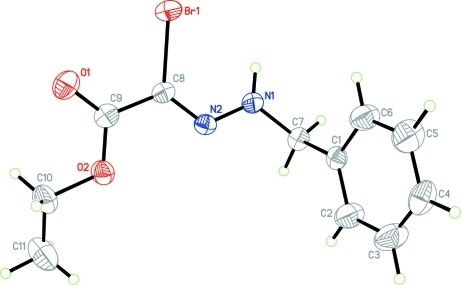
The structure of (I) showing 30% probability displacement ellipsoids.

**Fig. 2.**
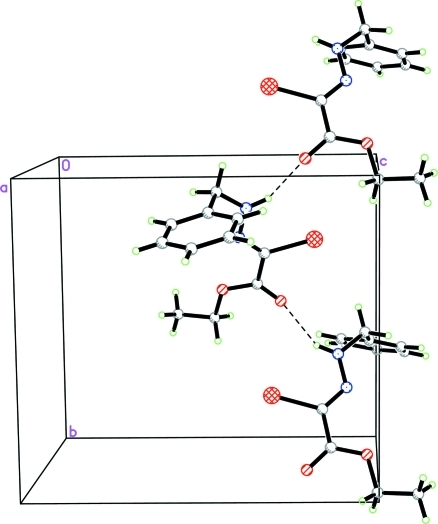
A view of the crystal structure of (I) showing chain to the b linked via N—H···O contact.

## supplementary crystallographic information

### Comment

(Benzylhydrazono)acetate is a key intermediate in the preparation for pyrazoline compounds (Bach *et al.*, 1994), which are selective for the NMDA receptors and show weak antagonists. In addition, it plays an important role in the synthesis of thiadiazole nucleus (Feddouli *et al.*, 2004; Abouricha *et al.*, 2005), which have been exhibited potential anti-inflammatory and analgesic activities (Hafez *et al.*, 2008). Herein, the structure of ethyl 2-bromo-(*Z*)-2-(2-benzylhydrazono)acetate has been determined.

The crystal structure of the title compound is given in Fig. 1. In the crystal, the adjacent molecules are stabilized by N—H···O hydrogen bonding, with the distance of 2.965 (4) Å (Table 1). Molecules are linked into chain along the *b* axis by the above hydrogen bond (Fig. 2).

### Experimental

To a stirred solution of ethyl 2,2-diethoxyacetate (1 ml, 5.6 mmol) and acetyl chloride (0.8 ml, 11.2 mmol) was added iodine (3 mg, 0.01 mmol). After the mixture was stirred for overnight, excess acetyl chloride was removed *in vacuo*, the residue in 1,4-dioxane (25 ml) was treated with benzylhydrazine dihydrochloride (1.09 g, 5.6 mmol) in water (10 ml), then the mixture was adjusted to pH 4. After 1 h the mixture was neutralized to pH 8 with saturated NaOH and evaporated in vacuo. The residue was added water and extracted with CH2Cl2, the organic layer was dried over MgSO4, filtered and concentrated. The crude compound was dissolved in AcOEt (8 ml), which was reacted with NBS (1.1 g, 6.2 mmol) for 2 h. After evaporation of the solvent, the residue was dissolved in CH2Cl2 and filtered, the filtrate was concentrated and purified by column chromatography (eluent: PE/AcOEt = 28/1) to give the title compound (0.67 g, 2.35 mmol) as a white solid. Colorless blocks of (I) were grown in PE/AcOEt (14/0.5, V/V) solution by slow evaporation at room temperature.

### Refinement

All H-atoms were positioned geometrically and refined using a riding model, with C—H = 0.96 Å (methyl), 0.97 Å (methenyl), 0.93 Å (aromatic), and *U*_iso_(H) =1.2*U*_eq_(C).

### Crystal data

**Table:**

C_11_H_13_BrN_2_O_2_	*F*(000) = 576
*M**_r_* = 285.14	*D*_x_ = 1.514 Mg m^−^^3^
Monoclinic, *P*2_1_/*c*	Mo *K*α radiation, λ = 0.71073 Å
Hall symbol: -P 2ybc	Cell parameters from 1479 reflections
*a* = 9.046 (1) Å	θ = 2.3–24.8°
*b* = 11.235 (1) Å	µ = 3.27 mm^−^^1^
*c* = 12.326 (2) Å	*T* = 294 K
β = 92.935 (4)°	Block, colorless
*V* = 1251.1 (3) Å^3^	0.25 × 0.14 × 0.07 mm
*Z* = 4	

### Data collection

**Table:**

Bruker APEX CCD diffractometer	2188 independent reflections
Radiation source: fine-focus sealed tube	1475 reflections with *I* > 2σ(*I*)
graphite	*R*_int_ = 0.019
phi and ω scans	θ_max_ = 25.0°, θ_min_ = 2.3°
Absorption correction: multi-scan (*SADABS*; Siemens, 1996)	*h* = −10→10
*T*_min_ = 0.495, *T*_max_ = 0.803	*k* = −13→7
4952 measured reflections	*l* = −7→14

### Refinement

**Table:**

Refinement on *F*^2^	Primary atom site location: structure-invariant direct methods
Least-squares matrix: full	Secondary atom site location: difference Fourier map
*R*[*F*^2^ > 2σ(*F*^2^)] = 0.038	Hydrogen site location: inferred from neighbouring sites
*wR*(*F*^2^) = 0.111	H-atom parameters constrained
*S* = 1.02	*w* = 1/[σ^2^(*F*_o_^2^) + (0.0656*P*)^2^] where *P* = (*F*_o_^2^ + 2*F*_c_^2^)/3
2188 reflections	(Δ/σ)_max_ < 0.001
146 parameters	Δρ_max_ = 0.26 e Å^−^^3^
0 restraints	Δρ_min_ = −0.61 e Å^−^^3^

### Special details

**Table:**

Geometry. All e.s.d.'s (except the e.s.d. in the dihedral angle between two l.s. planes) are estimated using the full covariance matrix. The cell e.s.d.'s are taken into account individually in the estimation of e.s.d.'s in distances, angles and torsion angles; correlations between e.s.d.'s in cell parameters are only used when they are defined by crystal symmetry. An approximate (isotropic) treatment of cell e.s.d.'s is used for estimating e.s.d.'s involving l.s. planes.
Refinement. Refinement of *F*^2^ against ALL reflections. The weighted *R*-factor *wR* and goodness of fit *S* are based on *F*^2^, conventional *R*-factors *R* are based on *F*, with *F* set to zero for negative *F*^2^. The threshold expression of *F*^2^ > σ(*F*^2^) is used only for calculating *R*-factors(gt) *etc*. and is not relevant to the choice of reflections for refinement. *R*-factors based on *F*^2^ are statistically about twice as large as those based on *F*, and *R*- factors based on ALL data will be even larger.

### Fractional atomic coordinates and isotropic or equivalent isotropic displacement parameters (Å^2^)

**Table:**

	*x*	*y*	*z*	*U*_iso_*/*U*_eq_	
Br1	0.44688 (4)	0.25237 (4)	0.31304 (3)	0.0668 (2)	
O1	0.3161 (3)	0.0317 (2)	0.2073 (2)	0.0755 (8)	
O2	0.3458 (2)	0.0749 (2)	0.03230 (18)	0.0534 (6)	
N1	0.5769 (3)	0.3675 (3)	0.1176 (2)	0.0501 (7)	
H1	0.5721	0.3973	0.1815	0.060*	
N2	0.5071 (3)	0.2673 (2)	0.0922 (2)	0.0424 (7)	
C1	0.8144 (3)	0.3760 (3)	0.0231 (3)	0.0436 (8)	
C2	0.8605 (4)	0.3447 (4)	−0.0771 (3)	0.0726 (11)	
H2	0.7946	0.3505	−0.1374	0.087*	
C3	1.0035 (5)	0.3045 (4)	−0.0906 (4)	0.0852 (13)	
H3	1.0321	0.2834	−0.1594	0.102*	
C4	1.1013 (4)	0.2958 (4)	−0.0045 (4)	0.0696 (11)	
H4	1.1976	0.2700	−0.0136	0.083*	
C5	1.0569 (4)	0.3253 (4)	0.0954 (4)	0.0830 (13)	
H5	1.1234	0.3182	0.1551	0.100*	
C6	0.9142 (4)	0.3659 (4)	0.1105 (3)	0.0721 (11)	
H6	0.8863	0.3862	0.1797	0.087*	
C7	0.6617 (3)	0.4268 (3)	0.0367 (3)	0.0475 (8)	
H7A	0.6717	0.5101	0.0563	0.057*	
H7B	0.6064	0.4228	−0.0327	0.057*	
C8	0.4424 (3)	0.2069 (3)	0.1640 (3)	0.0432 (7)	
C9	0.3625 (3)	0.0958 (3)	0.1382 (3)	0.0508 (8)	
C10	0.2617 (4)	−0.0317 (4)	0.0018 (3)	0.0722 (11)	
H10A	0.1758	−0.0378	0.0454	0.087*	
H10B	0.3226	−0.1017	0.0151	0.087*	
C11	0.2142 (5)	−0.0254 (4)	−0.1139 (3)	0.0950 (15)	
H11A	0.1489	0.0412	−0.1259	0.142*	
H11B	0.1632	−0.0974	−0.1349	0.142*	
H11C	0.2993	−0.0159	−0.1565	0.142*	

### Atomic displacement parameters (Å^2^)

**Table:**

	*U*^11^	*U*^22^	*U*^33^	*U*^12^	*U*^13^	*U*^23^
Br1	0.0724 (3)	0.0882 (4)	0.0405 (3)	−0.00890 (18)	0.00985 (18)	−0.00325 (18)
O1	0.1002 (19)	0.0639 (19)	0.0630 (17)	−0.0140 (15)	0.0091 (14)	0.0199 (14)
O2	0.0622 (13)	0.0418 (15)	0.0561 (14)	−0.0035 (11)	0.0025 (11)	−0.0015 (11)
N1	0.0508 (14)	0.0531 (19)	0.0473 (16)	−0.0048 (13)	0.0106 (12)	−0.0090 (14)
N2	0.0409 (13)	0.0448 (19)	0.0414 (15)	0.0058 (12)	0.0014 (12)	−0.0029 (12)
C1	0.0470 (16)	0.0314 (19)	0.053 (2)	−0.0016 (13)	0.0059 (15)	0.0020 (16)
C2	0.063 (2)	0.099 (3)	0.056 (2)	0.012 (2)	0.0053 (18)	0.000 (2)
C3	0.080 (3)	0.104 (4)	0.074 (3)	0.021 (3)	0.024 (2)	−0.012 (3)
C4	0.055 (2)	0.057 (3)	0.099 (4)	0.0130 (18)	0.022 (2)	0.011 (2)
C5	0.059 (2)	0.110 (4)	0.079 (3)	0.022 (2)	−0.005 (2)	0.015 (3)
C6	0.061 (2)	0.099 (3)	0.056 (2)	0.013 (2)	0.0058 (19)	0.001 (2)
C7	0.0470 (17)	0.041 (2)	0.055 (2)	0.0042 (14)	0.0064 (15)	0.0036 (16)
C8	0.0423 (16)	0.048 (2)	0.0393 (18)	0.0079 (14)	0.0031 (14)	0.0039 (15)
C9	0.0520 (18)	0.049 (2)	0.051 (2)	0.0064 (15)	0.0036 (15)	0.0049 (18)
C10	0.090 (3)	0.046 (3)	0.080 (3)	−0.014 (2)	0.003 (2)	−0.009 (2)
C11	0.114 (4)	0.069 (3)	0.099 (4)	−0.014 (3)	−0.026 (3)	−0.008 (3)

### Geometric parameters (Å, °)

**Table:**

Br1—C8	1.906 (3)	C4—C5	1.355 (6)
O1—C9	1.207 (4)	C4—H4	0.9300
O2—C9	1.328 (4)	C5—C6	1.391 (5)
O2—C10	1.457 (4)	C5—H5	0.9300
N1—N2	1.320 (3)	C6—H6	0.9300
N1—C7	1.451 (4)	C7—H7A	0.9700
N1—H1	0.8600	C7—H7B	0.9700
N2—C8	1.280 (4)	C8—C9	1.468 (5)
C1—C2	1.370 (4)	C10—C11	1.470 (5)
C1—C6	1.374 (5)	C10—H10A	0.9700
C1—C7	1.512 (4)	C10—H10B	0.9700
C2—C3	1.388 (5)	C11—H11A	0.9600
C2—H2	0.9300	C11—H11B	0.9600
C3—C4	1.350 (6)	C11—H11C	0.9600
C3—H3	0.9300		
			
C9—O2—C10	115.5 (3)	N1—C7—H7A	108.5
N2—N1—C7	119.5 (3)	C1—C7—H7A	108.5
N2—N1—H1	120.2	N1—C7—H7B	108.5
C7—N1—H1	120.2	C1—C7—H7B	108.5
C8—N2—N1	121.2 (3)	H7A—C7—H7B	107.5
C2—C1—C6	117.9 (3)	N2—C8—C9	122.6 (3)
C2—C1—C7	121.2 (3)	N2—C8—Br1	122.4 (3)
C6—C1—C7	120.7 (3)	C9—C8—Br1	115.0 (2)
C1—C2—C3	121.3 (4)	O1—C9—O2	124.2 (3)
C1—C2—H2	119.4	O1—C9—C8	122.7 (3)
C3—C2—H2	119.4	O2—C9—C8	113.1 (3)
C4—C3—C2	120.5 (4)	O2—C10—C11	109.5 (3)
C4—C3—H3	119.7	O2—C10—H10A	109.8
C2—C3—H3	119.7	C11—C10—H10A	109.8
C3—C4—C5	118.9 (4)	O2—C10—H10B	109.8
C3—C4—H4	120.6	C11—C10—H10B	109.8
C5—C4—H4	120.6	H10A—C10—H10B	108.2
C4—C5—C6	121.5 (4)	C10—C11—H11A	109.5
C4—C5—H5	119.3	C10—C11—H11B	109.5
C6—C5—H5	119.3	H11A—C11—H11B	109.5
C1—C6—C5	119.9 (4)	C10—C11—H11C	109.5
C1—C6—H6	120.1	H11A—C11—H11C	109.5
C5—C6—H6	120.1	H11B—C11—H11C	109.5
N1—C7—C1	114.9 (3)		

### Hydrogen-bond geometry (Å, °)

**Table:**

*D*—H···*A*	*D*—H	H···*A*	*D*···*A*	*D*—H···*A*
N1—H1···O1^i^	0.86	2.24	2.965 (4)	141

Symmetry codes: (i) −*x*+1, *y*+1/2, −*z*+1/2.
